# Therapeutic role of curcumin and its novel formulations in gynecological cancers

**DOI:** 10.1186/s13048-020-00731-7

**Published:** 2020-11-04

**Authors:** Mohammad Hossein Pourhanifeh, Maryam Darvish, Javad Tabatabaeian, Mahboobeh Rabbani Fard, Reza Mottaghi, Mohammad Javad Azadchehr, Moghaddaseh Jahanshahi, Amirhossein Sahebkar, Hamed Mirzaei

**Affiliations:** 1 Halal Research Center of IRI, FDA, Tehran, Iran; 2 Department of Medical Biotechnology, Faculty of Medicine, Arak University of Medical Science, Arak, Iran; 3 Faculty of Member, Ardestan Branch, Islamic Azad University, Ardestan, Iran; 4 Department of Oral and Maxillofacial Surgery, Kashan University of Medical Sciences, Kashan, Iran; 5 Trauma Research Center, Kashan University of Medical Sciences, Kashan, Iran; 6 Clinical Research Development Center (CRDC), Sayad Shirazi Hospital, Golestan University of Medical Sciences, Gorgan, Iran; 7 Neurogenic Inflammation Research Center, Mashhad University of Medical Sciences, Mashhad, Iran; 8 School of Pharmacy, Mashhad University of Medical Sciences, Mashhad, Iran; 9 Research Center for Biochemistry and Nutrition in Metabolic Diseases, Institute for Basic Sciences, Kashan University of Medical Sciences, Kashan, Iran

**Keywords:** Curcumin, Nanocurcumin, Endometrial cancer, Ovarian cancer, Cervical cancer, Curcumin derivatives, Natural compound

## Abstract

Gynecological cancers are among the leading causes of cancer-associated mortality worldwide. While the number of cases are rising, current therapeutic approaches are not efficient enough. There are considerable side-effects as well as treatment resistant types. In addition, which all make the treatment complicated for afflicted cases. Therefore, in order to improve efficacy of the treatment process and patients’ quality of life, searching for novel adjuvant treatments is highly warranted. Curcumin, a promising natural compound, is endowed with numerous therapeutic potentials including significant anticancer effects. Recently, various investigations have demonstrated the anticancer effects of curcumin and its novel analogues on gynecological cancers. Moreover, novel formulations of curcumin have resulted in further propitious effects. This review discusses these studies and highlights the possible underlying mechanisms of the observed effects.

## Background

Natural compounds like curcumin, epigallocatechin gallate (EGCG), quersetin, and resveratrol, have been shown to modulate some genetic and epigenetic mechanisms, which may increase sensitivity of cancer cells to conventional agents and thus inhibit tumor growth [1, 2]. Curcumin, the main bioactive ingredient of turmeric, has been revealed to possess various therapeutic potentials, such as anti-tumor [3, 4], anti-atherosclerotic [5, 6], anti-microbial [7], anti-oxidant [8], and anti-inflammatory [9–11]. A wide variety of molecular targets have been reported for curcumin, including some transcription factors, gene modulators, kinases, growth factors, and cell membrane receptors [12–16]. Due to its various and pleiotropic functions, increasing number of studies has been focused on curcumin, specifically in malignancies.

Numerous investigations have shown that curcumin acts as a beneficial adjuvant and chemopreventive agent for cancer [17]. Notably, human studies have demonstrated the tolerability, safety and anti-carcinogenic ability of this compound for humans [13, 14]. The promising outcome of these studies has helped to raise the hope for a brighter future in cancer treatment.

Gynecologic cancers include an unlimited and abnormal cell growth, developing in female reproductive system, such as endometrial, ovarian, cervical, primary peritoneal, vulvar and vaginal malignancies. According to reports, more than 1 million patients were newly diagnosed, and more than 580 000 mortalities occurred because of endometrial, cervical, and ovary cancers in 2018 [15]. While endometrial and cervical cancers are diagnosed in early stages, ovarian cancer is usually diagnosed in more advanced stages, when treatment is more challenging [16, 18]. Despite all the developments in cancer therapy and emergence of novel treatments, these cancers still have considerable mortality rates. Therefore, Therefore, introducing novel agents which can potentially improve the therapeutic outcome for these affected patients is highly needed. Here, in this review we focus on current evidence for the efficacy of curcumin and its novel formulations in gynecological cancers.

## The role of curcumin in gynecological cancers

In terms of gynecological cancers, curcumin has been shown to modify the effects of risk factors during the course of cancer progression and since the very beginning. Some of those well-known risk factors are obesity, smoking, estrogen and human papillomavirus (HPV) infections [19, 20]. Interestingly, curcumin has been shown to reduce estrogen synthesize, estrogen-derived DNA damage, inflammation in the adipose tissue, and inhibit E6 and E7 onco-protein expression in HPV [19].

According to in vitro studies, curcumin can prevent invasion and migration of endometrial carcinoma (EC) cell and plays an anti-metastatic role due to a reduction in the production and fuction of matrix metalloproteinases-2 and -9 (MMP-2 and MMP-9). Degradation of tumor extracellular matrix by these enzymes is a possible cause of metastasis and invasion of myometrial cancer to lymph node in type II EC. curcumin has been shown to suppress the ERK signaling pathway and decrease the expression of these enzymes [21].

Curcumin has been reported to induce apoptosis in ovarian cancer cells, through P53-independent pathway, but similar to wild-type P53 cells. Curcumin-treated HEY cells showed poly (ADP-ribose) polymerase-1 cleavage, DNA fragmentation, nuclear fragmentation and condensation, indicating the induction of cell apoptosis. Moreover, curcumin is able to induce apoptosis through both intrinsic and extrinsic mechanisms. The expression of antiapoptotic regulators, survivin and Bcl-2, was decreased following an increase in the activity of p38 protein, mitogen-activated protein kinases (MAPK). The anticancer cell death caused by curcumin was reported to also occur through suppressing the prosurvival Akt signaling in different ovarian cancer cells [22]. The expression of urokinase-type plasminogen activator (uPA) was reportedly blocked by curcumin in highly invasive human ovarian cancer cell line, HRA, which has a role in cancer metastasis. uPA is a serine protease expressed via Src-MAPK/ERK-AP-1 and Src-MAPK/ERK-PI3K/ Akt-NF-kB pathways in response to TGF-B1, where curcumin inhibits the AP-1 complex formation [22]. Accordingly, curcumin can potentially improve the outcome in advanced gynecological cancers impeding cancer invasion and progression [23]. In the following sections, we discuss recent investigations on curcumin therapy for endometrial, cervical and ovarian cancer.

### Curcumin and endometrial cancer

Endometrial cancer (EC), the most frequent malignant tumor of the genital tract in females, occurs in peri- and postmenopausal women [24, 25]. It is estimated that more than 50,000 new cases were diagnosed, and about 8,500 cases were expired due to this malignancy in the US in 2014. Recently, due to the life style changes, gynecological cancers are causing increasing mortalities [26]. The majority of affected individuals are diagnosed early and usually cured with surgery alone or in combination with radiatiotherapy [27]. However, mortality rate of this cancer has experienced a sharper rise than its incidence over the last decades [28–31]. Despite all the development in therapeutic strategies for this malignancy, end-stage patients face poor prognoses, and the 5-year survival rate is as low as 25–45% [26]. Additionally, the current therapeutic approaches are not effective for 15% of patients who show aggressive phenotype [27].

It was reported that in a 30-day *in vivo* investigation, daily intraperitoneal administration of 50 mg/kg curcumin decreased the volume of tumor five-fold in comparison with that in vehicle-treated animals [32]. Sirohi and his colleagues also carried out in vitro experiments utilizing both HEC-1B and Ishikawa cells and indicated that curcumin exhibits its anti-migratory impacts *via* increasing the Slit2 expression [32]. Slit2 induction downregulated the expression of migratory proteins, including the matrix metalloproteases MMP2/9, stromal cell-derived factor-1 (SDF-1), and CXCR4 (chemokine receptor 4) in EC cells [32]. This study indicated that curcumin suppresses tumor growth and inhibits proliferation of EC cells [32]. It also mediates ROS-induced apoptosis and shows inhibitory effects on migration of Hec-1B and Ishikawa cells through Slit-2-induced downregulation of MMP-2 and -9, SDF-1, and CXCR4 [32].

Androgen receptors (ARs) are ligand-dependent nuclear transcription factors which have been related to various tumors types, including endometrial, liver, bladder, and prostate tumors [33]. It has been demonstrated that curcumin dose- and time-dependently increases apoptosis and blocks proliferation of EC cell line RL-952 [34]. Curcumin also targeted Wnt pathway, which plays a crucial role in tumor proliferation and progression, and subsequently declined the expression of AR in cancer cells [34]. In a research carried out by Chen et al. [21], it was shown that curcumin inhibits the invasion and migration of endometrial carcinoma cells. Additionally, it decreased the proteinase activity and matrix metalloproteinase (MMP)-2 and -9 expression [21]. Curcumin in combination with ERK inhibitor U0126 synergistically reduced the expression of MMP-2/-9, leading to inhibit the cancer cells invasion [35]. Sun et al. illustrated that, in addition to suppression of EC cell invasion via downregulating MMP-2, curcumin inhibits their proliferation as well [35]. Feng et al. revealed that curcumin suppresses the apoptosis and proliferation of human endometrial carcinoma cells through downregulating the expression of AR *via* Wnt pathway [34]. Table 1 shows the available data about the effects of curcumin in EC therapy.

**Table 1:** Performed studies on curcumin and EC therapy

Type of curcumin	Dose	Target(s)	Effect(s)	Type of cell line	Ref
Curcumin	6 μM	CTGF, MMP-2, -9 Slit-2, CXCR4, SDF-1, MMP9	Induction of apoptosis Inhibition of tumor proliferation, migration, invasion, and growth	Ishikawa Hec-1B	[32]
	6 μM for 48h	-	Induction of ROS production	Ishikawa cell	[32]
	50 mg/kg	-	Reduction the tumor volume in mice	-	[32]
	30 μmol/L	MMP-2	Curcumin can suppress invasion and proliferation of endometrial cancer cell	Ishikawa	[35]
	50 μM	STAT-3 PIAS-3	Curcumin can inhibit JAK-STAT signaling through PIAS-3 activation	RL95-2 Ishikawa OVCA cell	[36]
	30 μM for 120h	TREK1	Curcumin has an antiproliferative effect on endometrial cells.	Ishikawa	[37]
	30 μM	MMP-2, -9 ERK	Curcumin can suppress invasion and proliferation of endometrial cancer cells through inhibition of MMP-2 and MMP-9 and the ERK signaling pathway	HEC-1B	[21]
	300 mg/kg.d	Bcl-2	Curcumin inhibits the Bcl-2 expression	-	[38]
	40 to 60 μM for 3h	Ets-1 Bcl-2	Curcumin can decrease the Bcl-2 and Ets-1 expression and induces apoptosis.	HEC-1-A	[39]
	100 μM/L	Androgen receptor (AR)	Curcumin can suppress apoptosis and proliferation of endometrial cancer cells through decreasing expression of androgen receptor	NA	[34]
	112.5 μM	-	Curcumin has an antiproliferative effect on MCF-7, MG-63 and MDA-MB-231 cells	MDA-MB-231 MCF-7 MG-63	[40]
Curcumin loaded amphiphilic mixed micelles	10 μM	Survivin Bcl-2 PARP	Inhibition of tumor growth, Apoptosis induction	Ishikawa	[41]
Liposomal Curcumin	NA	NF-κB	Inhibition of tumor growth	Ishikawa HEC-1	[42]
Curcumin Phytosome	2 g/day	-	Immunomodulatory effects	-	[43]

### Curcumin and cervical cancer

Cervical cancer is the third most prevalent malignancy and the fourth leading cause of cancer-related mortality in women [44]. Nowadays, its incidence is triggering the younger females [45]. Patients who are still at early stages have good prognosis and successfully respond to conventional treatments, including chemoradiation and/or surgery [46]. While these standard therapies could cause serious damages to vaginal and ovarian functions [47], patients with extrapelvic ingagement have 5-year survival rate of 17%, and in women with recurrent disease this rate is less than 5% [48]. Human papilloma virus (HPV) infection is the initial step in most of the cervical cancer cases [49]. In addition to chronic infection with HPV, smoke carcinogen (benzo [a]pyrene) and cigarette smoking are considered as major risk factors related to cervical cancer [50, 51].

Curcumin has revealed concentration-dependent chemotherapeutic and chemopreventive impacts in a number of investigations [52]. Curcumin exerts cytotoxic effects in cervical cancer cells in a time- and dose-dependent approach, especially in HPV-infected cells [53]. It has been confirmed that, *via* selectively suppressing activator protein 1 (AP-1) activity, curcumin downregulates HPV18 transcription, which reverses the fra-1 and c-fos expression dynamics in cervical cancer cells [53]. Higher suppressive function of curcumin against cervical cancer is because of the inhibition of the mitochondrial pathway, iNOS, COX-2, and cyclin D1 activity, and ERK and Ras signaling pathways as well as telomerase action [54, 55]. Remarkably, curcumin acts through targeting several signaling pathways, making the proliferation of cervical cancer cells revert to normal. It also mediates important alterations in tumor-associated proteins correlated with cell metabolism, cell cycle, and carcinogenicity in HeLa cells [56].

He and co-workers reported that both curcumin-photodynamic therapy (PDT) and dual antiplatelet therapy (DAPT), a Notch receptor blocker, are capable of inducing apoptosis and blocking the proliferation of cervical cancer Me180 cells [57]. Moreover, DAPT has synergistic effects on curcumin-PDT in cervical cancer treatment, which is primarily associated with NF-κB and Notch-1 down-regulation [57]. Ghasemi et al. recently performed an in vitro study to evaluate the probable mechanisms related to anticancer effects of curcumin on cervical cancer cell line [58]. They revealed that curcumin suppresses proliferation and invasion of cervical cancer cells through Wnt/β-catenin and NF-kB pathways impairment [58]. Shang and colleagues reported that, 13 μM curcumin induces cell death in HeLa cells via induction of DNA damage and chromatin condensation [59]. It has been demonstrated that curcumin in combination with ultrasound exerts more beneficial effects against cervical cancer cells [60]. Carr et al. indicated that this combination enhanced apoptosis in SiHa or HeLa cells [60]. Curcumin alone caused less necrosis compared to the combination therapy. They showed the ultrasound capacity to elevate curcumin effectiveness [60]. Recently, it has been reported that curcumin elevates intracellular ROS levels in cervical cancer cells, but not in healthy epithelial cells [61]. This effect leads to inhibition of ER stress and partly restoration of the viability in cancer cells treated with curcumin [61]. Collectively, these observations show that curcumin promotes ER stress-mediated apoptosis in cervical cancer cells through increasing the cell type-specific ROS generation [61]. According to Yoysungnoen-Chintana's report, curcumin at high doses (1,000 and 1,500 mg/kg) inhibits angiogenesis as well as tumor growth in CaSki-implanted mice possibly induced *via* downregulating the EGFR, COX-2 and VEGF expression [62]. Table 2 shows the available findings on curcumin therapy for cervical cancer *in vivo* and *in vitro*.

**Table 2:** Conducted investigations on the treatment of cervical cancer with curcumin

Type of curcumin	Dose	Main target (s)	Main effect (s)	Model (in vivo/in vitro/human)	Cell line	Ref
Curcumin	20 μM for 72h	N-cadherin, Vimentin, Slug, PIR, Pirin	Inhibition of cancer cell growth, migration, invasion Inhibition of angiogenesis Induction of apoptosis and necrosis Induction of cell cycle arrest Increased radiosensitization of cancer cells	In vitro	SiHa	[63]
	2.5, 5 μmol/L In vivo (150-200 μL)	Notch-1, NF-κB, VEGF		In vivo In vitro	Me180	[57]
	5× IC50 (34.23 μM/ml)	Wnt/β-catenin NF-kB pathway		In vitro	HeLa	[64]
	13 μM	BRCA1, p-p53, p-H2A.XSer140		In vitro	HeLa	[59]
	IC50= 16.52 μM	ROS, p21, Bax, p53, ROS, p21, Bax		In vitro	HeLa	[65]
	10 μM In vivo (4 mg/kg)	-		In vitro In vivo	HeLa	[66]
	10 μM	TGF-β activates Wnt/β-catenin signaling pathway		In vitro	SiHa HeLa	[67]
	10 μM	NF-κB-p53-caspase-3 pathway	Curcumin improves the paclitaxel-induced apoptosis of cervical cancer cell lines infected with HPV.	In vitro	CaSki HeLa	[68]
	5 μM	-	Curcumin-induced apoptosis and oxidative stress	In vitro	HeLa	[69]
	1000 and 1500 mg/kg for 30 days	-	Curcumin inhibits angiogenesis and tumor growth mediated by decreasing the expression of VEGF, EGFR, and COX-2.	In vivo	-	[62]
	50 μM	-	Curcumin sensitizes cervical cancer cells to cisplatin-based chemotherapy through inhibition of Pgp1and MRP1.	In vitro	SiHa SiHaR	[70]
	20 μM	-	Curcumin induced ER stress-mediated apoptosis via increasing of ROS generation and by activation of CHOP	In vitro	C33A CaSki HeLa ME180	[61]
	IC50: 17 μM (HeLa), 12 μM (ME-180), 51 μM (SiHa), 21 μM (SW756) Dose: 50 μM for 48h	-	Curcumin-based vaginal cream effectively eradicates HPV positive cervical cancer cells.	In vitro	HeLa ME-180 SiHa SW756	[71]
	10 and 25 μM	Akt, MAPK, and AP-1 pathways	Curcumin potentiates the antitumor effects of paclitaxel by downregulating Akt, MAPK, and AP-1 pathways and decreasing the transcription of NF-kB target genes.	In vivo	-	[72]
	25 and 50 μM	-	Curcumin can induce apoptosis by inhibition of PCNA, Cyclin D1, telomerase, and p16 and by activation of p53 and p73 in HPV-negative cancer cells pretreated with estradiol.	In vivo	HeLa SiHa CaSki C33A	[54]
	50 and 100 μM for 24h	Apoptosis and inflammatory pathways	Curcumin mediates apoptosis in SiHa and HeLa cell lines. Curcumin can act as an anti-proliferative and anti-inflammatory agent for Ca Ski, HeLa, and SiHa cells	In vitro	HeLa SiHa CaSki	[73]
	15 μM for 48h	-	Curcumin exhibits antitumor activity against cervical cancer cells. Curcumin downregulates PGE2 expression.	In vitro	HeLa	[56]
	10 μM for 8h	MAP kinase pathway	Curcumin is a potent radiosensitizer by increasing ROS production and overacts the MAP kinase pathway.	In vitro	HeLa SiHa	[74]
	10μMCombined curcumin (10μM) ultrasound (8 s of 5-7.5 MHz)	-	Curcumin can lead to necrosis in cervical cancer cell lines. Combined curcumin ultrasound enhances necrosis in cervical cancer cell lines.	In vitro	HeLa SiHa C33A	[60]
ST06-AgNPs	IC50: 1μM Dose: 1-2 μM Dose: 5 mg/kg body weight for 30 days (In vivo)	-	Inhibited cancer cell growth	In vivo In vitro	HeLa	[75]
Folic acid-modified liposomal curcumin	IC50: 1.47 μg/mL for free curcumin IC50: 0.45 μg/mL for (DSPE)-PEG2000-FA-LPs/CUR Dose: 25 mg/kg for 51 days (In vivo)	-	Anti-proliferative effects	In vitro In vivo	HeLa	[76]
4-Bromo-4'-chloro pyrazoline	IC_50:_ 8.7μg/ml for Chloro bromo analogIC_50_: 42.24 μg/mL for curcumin	-	Apoptosis induction	In vitro	HeLa	[77]
Chloro and bromo-pyrazolecurcumin	IC50: 14.2 and 18.6 μg/ml for Chloro derivative and bromo analog, respectively. IC50: 42.4 μg/ml for curcumin	-	Apoptosis induction	In vitro	HeLa	[78]
Curcumin-loaded microbubble	1.25–40 μM	-	Decreased cancer cell viability	In vitro	HeLa	[79]
Bisdemethoxycurcumin	5μM for 24 and 48h	NF-kB, MMP-2 and -9 Pathways	Anti-migration and anti-invasion effects	In vitro	HeLa	[80]
Curcumin-PDT	-	Notch signaling pathway	Necrosis induction	In vivo	Me180	[81]
Curcumin-loaded micells	50 μg/mL	-	Increased cytotoxicity against cancer cells Apoptosis induction	In vitro	HeLa HepG2 NIH-3T3	[82]
Demethoxycurcumin	15 μM IC_20_: 7.5 μM	NF-κB Pathways	Anti-migration and anti-invasion effects	In vitro	HeLa	[83]
Curcumin-loaded chitosan nanoparticles	24μM	-	Apoptosis induction Anti-proliferative effects Showed better chemopreventive and chemotherapeutic effects than curcumin	In vitro	SiHa	[84]
Difluorinated curcumin Folate decorated bovine serum albumin (FA-BSA) nanoparticles loaded with Difluorinated curcumin (CDF) (FA-BSA-CDF)	Dose: 2 μM (Difluorinated curcumin and FA-BSA-CDF) Dose: 0.5 μM (Combination)	-	Synergistic anticancer effectsApoptosis induction	In vitro	HeLa SKOV3	[85]
Curcumin-nanoemulsion	20 to 40*μ*M	-	Apoptosis induction	In vitro	CasKi SiHa HaCaT	[86]
Curcumin-Loaded TPGS/F127/P123 Mixed Polymeric Micelles	Dose: 8 μg/mL Dose: 25 mg/kg for 11 times in 2 days (In vivo)	-	Increased cytotoxicity against cancer cells Induction of apoptosis and cell cycle arrest	In vivo In vitro	HeLa NIH3T3 cells	[87]
Curcumin-loaded chitosan-alginate-sodium tripolyphosphate nanoparticles	50 μg/mL	*Bax , Bcl-2*	Anti-proliferative effects Apoptosis induction	In vitro	HeLa	[88]
Folic acid conjugated polymeric micelles loaded with a curcumindifluorinated	0.47 ± 0.14 μM	PTEN, NF-κB	Apoptosis induction	In vitro	HeLa	[89]
Curcumin-loaded chitosan Nanoparticles	108 μM	*Bax, Bcl-2*	Apoptosis induction	In vitro	SiHa Hela Caski C33a	[90]
Tetrahydrocurcumin	100, 300, or 500 mg/kg body weight for 30 days	COX-2, EGFR, p-ERK1&2, p-AKT, Ki-67	Apoptosis induction Antitumor Effect	In vivo	CaSki	[91]
Nano-Curcumin	20 and 25 μM for 48h	Anti-survival pathways	Inhibited cancer cell growth Induction of apoptosis and cycle cell arrest	In vitro	SiHa, Caski	[19]
Tetrahydrocurcumin	50, 100 mg/kg	-	Inhibited cancer cell growth Anti-angiogenesis effects	In vivo In vitro	CaSki	[92, 93]
Curcumin (CCM)-loaded nanoscale zeolitic imidazolate framework-8 (CCM@NZIF-8) nanoparticles	Dose: 1-10 μg/mL Dose: 2.5 mg/kg body weight for 6 times in 2 days (In vivo)	-	Anti-proliferative effects Showed higher efficacy than free curcumin	In vivo In vitro	HeLa	[94]
Curcumin-loaded cationic liposome	IC_50_: 16, 21 μM	-	Apoptosis induction	In vitro	HeLa SiHa	[95]

### Curcumin and ovarian cancer

In the western world, ovarian cancer leads to the greatest mortality rate among all the gynecologic cancers. Affected individuals are usually diagnosed too late at advanced stages, when the cancer has spread to the peritoneal surfaces [96–98]. Surgery is the most efficient therapy for advanced stages, and is proceeded by chemotherapy with paclitaxel and carboplatin. After three chemotherapy cycles, interval cytoreductive surgery is alternatively done [96–98].

Curcumin reduces the needed dose of radiation and cisplatin in growth suppression of cisplatin-resistant ovarian cancer cells [99]. Curcumin, in combination with low amounts of cisplatin, also promotes apoptosis [99]. The decrease of cisplatin resistance in ovarian cancer cells by curcumin is probably through regulating extracellular vesicle-induced transfer of miR-214 and maternally expressed 3 (MEG3) [100]. This polyphenol has cytotoxic effects against platinum-resistant OVCAR-3 cells, which can be significantly enhanced by the compound Y15 (1, 2, 4, 5-benzene tetra amine tetrahydrochloride) [101]. Moreover, it decreases the number and size of ovarian tumors through inhibiting STAT3 and NF-κB signaling and inducing nuclear factor erythroid 2/heme oxygenase1 (Nrf2/HO-1) pathway [102]. In addition, curcumin induces G2/M cell-cycle arrest in cisplatin-resistant ovarian cancer cells *via* increasing apoptosis and phosphorylation of p53 by caspase-3 activation followed by poly (ADP-ribose) polymerase-1 (PARP) degradation [103].

In human ovarian cancer cell lines A2780 and SK-OV-3, curcumin is able to induce apoptosis as well as protective autophagy *via* suppression of AKT/mTOR/p70S6K pathway, demonstrating the synergistic impacts of curcumin and autophagy suppression [104]. Curcumin inhibits the activity of Sarco/endoplasmic reticulum calcium ATPase (SERCA) leading to dysregulation in Ca^2+^ homeostasis; and hence, contributes to apoptosis in ovarian cancer cells [105]. As discussed before, curcumin has anti-proliferative effects and can restrict tumor growth by this potential. Shi and colleagues demonstrated, in addition to apoptosis induction, 40 μM curcumin suppressed the growth of ovarian cancer cells [106]. Lin et al. showed that, in addition to apoptosis induction and anti-proliferative effects, curcumin suppresses angiogenesis in ovarian cancer *in vivo* and *in vitro* [107]. Furthermore, curcumin is capable of suppressing endothelial growth factor (EGF)-mediated Aquaporin 3 upregulation and cell migration in CaOV3 ovarian cancer cells through its inhibitory impacts on EGFR and AKT/ERK activation [108]. In an in vitro investigation, Xiaoling et al. reported that curcumin inhibits the metastasis and invasion of the human ovarian cancer cells SKOV3 through suppressing CXCR4 and CXCL-12 expression [109]. Wahl and his colleagues illustrated that combined treatment of Apo2 ligand (Apo2L)/TNF-related *apoptosis*-inducing ligand and curcumin (5-15 μM) leads to increased apoptotic cell death induction [110]. They also stated that, because the mentioned combination are able to activate both the intrinsic and extrinsic apoptosis pathways, they may overcome chemoresistance to conventional chemotherapeutic drugs [110]. Epithelial ovarian cancer (EOC) spheroids have a key role in chemoresistance development [111]. A research was conducted to evaluate curcumin effects on chemoresistance and antiperitoneal metastasis in EOC spheroids [112]. It was indicated that high invasive EOC cells that form spheroids express a high level of aldehyde dehydrogenase 1 family member A1, a cancer stem cell marker, which was markedly downregulated by curcumin [112]. Curcumin significantly increased EOC spheroids’ sensitivity to cisplatin and abolished their sphere-forming ability [112]. Furthermore, curcumin inhibited pre-existed EOC spheroids’ growth and also suppressed their invasion to the mesothelial monolayers and their adhesion to extracellular matrix [112]. Table 3 summarizes the current data on the therapeutic effects of curcumin on ovarian cancer *in vivo* and *in vitro*.

**Table 3:** Recent studies on curcumin treatment for ovarian cancer

Type of curcumin	Dose	Main target (s)	Main effect (s)	Model (in vivo/in vitro/human)	Cell line	Ref
Curcumin	20 μM for 96 hours	Wnt/β-catenin	Inhibition of tumor growth, migration, and invasion Inhibition of epithelial-mesenchymal transition Inhibition of autophagy Induction of apoptosis Increased the sensitivity of cancer cells Induced cell cycle arrest Antioxidant and anti-proliferative effects	In vitro	SKOV3	[113]
	20 μM for 48 hours	-		In vitro	ES2, OVCAR3	[114]
	30, 40 μM for 48 hours	AKT/mTOR/p70S6K		In vitro	SK-OV-3, A2780	[104]
	400 μM	NQO1, *c-Myc*, *Cyclin B1*, *Cyclin D1*		In vitro	OVCAR3, OVCAR5, SKOV3	[115]
	20 μM	FAK		In vitro	SKOV-3, OVCAR-3, PA-1	[116]
	25.8, 53.0 mg/day	NF-κB		In vivo	-	[117]
	Dose: 20 mg/kg (In vivo) Dose: 10 μM for 48 hours	miR-124		In vitro In vivo	SKOV3	[118]
	1 μM for 36 hours	miR-214, MEG3		In vitro	A2780 OVCAR-3 SKOV3	[100]
	60 μM for 72 hours	*-*		In vitro	SKOV3	[119]
	50 μM	MMP-9, CD44, osteopontin		In vitro	SKOV3	[120]
	10 μM for 2 and 3 hours	STAT3, IL-6, IL-8		In vitro	PA-1, OVCAR-3	[121]
	80 μM for 24 hours	Caspase-3, PARP-1, Bcl-2, P13K/Akt, P38 MAPK		In vitro	HEY, OVCA429, OCC1, SKOV3	[22]
	50 μM for 240 min	Caspase-3, IL-6, STAT-3, p-JAK-1 and p-JAK-2, PIAS-3, SOCS-3		In vitro	OVCA420, OVCA429	[36]
	40 μM for 24 hours	AQP-3		In vitro	CaOV3	[108]
	0.5 μM for 48 hours	ROS, glutathione		In vitro	C13, 2008	[122]
	50μM for 24 hours	AMPK, p38, p53		In vitro	CaOV3	[123]
	40μM for 72 hours	PI3K/Akt		In vitro	SKOV3	[124]
	60μM	ALDH1A1		In vitro	SKOV3 OVCAR3	[112]
	15μM for 24 hours	SERCA		In vitro	MDAH 2774, SKOV3, PA1	[105]
	41.6μmol/L for 12 hours	Bcl-2, p53, MDM2, NFκB, caspase-3		In vitro	A2780	[125]
	3 μM for 12 hours	Rho A/Rho-kinase		In vitro	SKOV3	[126]
	40μM for 48 hours	Bcl-2, Bcl-xL, caspase-3, p53, Bax		In vitro	HO-8910	[106]
Curcumin-loaded biodegradable PLGA nanoparticles (CUR-NP)	0.1 mg/mL 50 μM	-	CUR-NP exhibited better physicochemical properties compared to free curcumin	In vitro	SK-OV-3	[127]
Curcumin-coated silver nanoparticles (cAgNPs)	2μg/mL for 48 hours	Caspase 3/9, p53, MPP-9	Apoptosis induction	In vitro	A2780	[128]
Combinational polymeric micelles for co-delivery of quercetin/resveratrol and resveratrol/curcumin	1 mg/ml	Caspase-3	Apoptosis induction	In vitro In vivo	ES2-Luc, A2780ADR	[129]
DNC	55 μM for 48 hours	LSINCT5, CCAT2, ABO73614, ANRIL, FAL1, BC200, MALAT1	Apoptosis induction Inhibited tumor growth	In vitro	OVCAR3 SKOV3	[130]
Curcumin-loaded PLGA MPs	Dose: 20 and 40μM for 48 and 72 hours Dose: 100 mg/kg (In vivo)	-	CPMs formulation was more effective than free curcumin in suppressing proliferation of ovarian cancer cells	In vitro In vivo	SKOV-3	[131]
Curcumin nanoparticle	50 μg/ml	**P-gp**	Decreased chemotherapy resistance Induced cell cycle arrest Apoptosis induction Antioxidant effects	In vitro	A2780	[132]
	6.62 μg/mL	HSP70		In vitro In vivo	SKOV3	[133]
Difluorinated curcumin Folate decorated bovine serum albumin (FA-BSA) nanoparticles loaded with Difluorinated curcumin (CDF) (FA-BSA-CDF)	162.8 nM	-	Apoptosis induction	In vitro	SKOV3	[85]
FA-SMA-CDF nanomicelles	1.55 ± 0.23 μM for 72 hours	PTEN, NFκB,	Apoptosis induction	In vitro	SKOV3	[89]
Demethoxycurcumin	20, 40 and 80 μM for 48 hours	IRS2/PI3K/Akt miR-551a	Anti-proliferative effects Apoptosis induction	In vitro	ES2, HO8640, HO8640PM, SKOV3	[134]
Doxorubicin/curcumin co-loaded alginate-shelled nanodroplets	-	-	Inhibited tumor growth	In vitro In vivo	A2780	[135]
Boron-curcumin complex	6 mg	-	Enhancement of anticancer effects of curcumin	In vitro	IGROV-1	[136]
Curcumin-loaded NLC Curcumin	30 μM for 24 hours	PARP, caspase-3	Apoptosis induction	In vitro	A2780	[137]
Curcumin-loaded δ-T3 nanoemulsion	1.96 ± 0.37 mg/ml	NF-κB	Anti-proliferative effects Apoptosis induction	In vitro	OVCAR-8	[138]
Bisdemethoxycurcumin	15 μM for 24 hours	MMP-2, -9 CD147, uPA, ICAM-1, VCAM-1, TIMP-1, NF-κB p65, VEGF	Inhibited growth, adhesion and motility of tumor cells Arrested cell cycle Anti-migration and anti-invasion effects Antioxidant effects	In vitro	SKOV-3	[139]
Monofunctional platinum (II) *tris* (quinoline) chloroplatinum (II)	60 to 200 μM for 72 hours	-	Greater toxicity on resistant tumor cells compared to cisplatin	In vitro	A2780	[140]
ASA/Cur-coloaded mPEG-PLGA nanoparticles	5 μg/mL	Caspase-3, -9 Bcl-2, Bax	Apoptosis induction The co-delivery of curcumin enhanced its antitumor activity	In vitro	ES-2, SKOV3	[141]
Curcumin and platinum-loaded micelles	1 mg	-	The co-delivery of curcumin enhanced its antitumor activity	In vitro	A2780	[142]
MPEG-PCL curcumin micelles	5 μg/mL	-	Induced cell cycle arrest and apoptosis	In vitro	A2780	[143]
Monofunctional platinum (II) complex *tris* (benzimidazole) chloroplatinum (II)	180 mg	-	Synergistic anticancer effects on cisplatin-resistant cancer cells	In vitro	A2780	[144]
Co-delivery of paclitaxel and curcumin by transferrin-targeted PEG-PE-based mixed micelles	20 μM 25 mg/kg	Annexin V	Apoptosis induction	In vitro In vivo	NCI-ADR-RES SK-OV-3	[145]
PEG-PE/vitamin E micelles for co-delivery of paclitaxel and curcumin	10 μM25 mg/kg	-	Showed synergistic effects compared to curcumin or paclitaxel alone against cancer cells	In vitro In vivo	SK-OV-3	[146]
Curcumin loaded poly(2-hydroxyethyl methacrylate) nanoparticles	10 μg/ml	NFkB, survivin, VEGF, COX-2	Anti-proliferative effectsNecrosis induction	In vitro	SK-OV-3	[147]
*B19 (1E, 4E)-1, 5-bis (2-methoxyphenyl) penta-1, 4-dien-3-one	10 μM for 12 hours	ER stress, UPR	Inhibited autophagy Apoptosis induction	In vitro	HO8910	[148]
Curcumin encapsulated Poloxamer 407/TPGS mixed micelles	-	P-gp	Increased cytotoxicity against multidrug resistant ovarian cancer cells	In vitro	NCI/ADR-RES	[149]

## Limitation of curcumin

The clinical anti-cancer effects of curcumin have not yet been fully documented despite its potential effects. Poor solubility in water is one of the main restrictions in curcumin application (only about 11 ng / ml), thereby possibly limiting its beneficial effects [150]. Other drawbacks are instability in alkaline and neutral environments, and low bioavailability because of rapid metabolism and elimination [151, 152].

Intravenous, peritoneal and oral administration of curcumin leads to the formation of glucuronide metabolites in the liver and excretion through the bile into the gastrointestinal tract [153, 154]. In a study, daily administration of curcumin (3600 mg) in patients with metastatic colorectal cancer, the circulatory curcumin content was in nanomolar level [155]. Similarly, other findings showed minor changes in the peripheral blood of patients at high risk following daily administration of curcumin (8000 mg) [156]. After the administration of curcumin at the doses of 500-8000 mg, there was no measurable level in the blood and only a small amount of its metabolites was measured in research units taking 10,000-12,000 mg [157, 158]. It can be concluded that the therapeutic potentials of curcumin may be enhanced by improving its bioavailability and solubility.

## Novel formulations of curcumin in the treatment of gynecological cancers

As mentioned before, curcumin has low absorption and poor bioavailability [159]; thus, several investigators have been attempting to improve its therapeutic effectiveness and pharmacokinetic profile by approaches such as development of new analogs [150, 160, 161]. Structural alterations in curcumin molecule leads to numerous beneficial properties for treating different diseases, including neurodegenerative diseases, diabetes, and cardiovascular diseases [159]. Curcumin and its analogs are extensively utilized as antioxidants, antimicrobial, anti-inflammatory, and anticancer agents. Successful attempts for synthesizing new analogues of curcumin with enhanced bioactivity have been reported [3, 162, 163]. It has been indicated that curcumin has anticancer functions due to its impact on various biological pathways implicated in metastasis, apoptosis, cell cycle regulation, tumorigenesis, oncogene expression, and mutagenesis [164, 165]. Recently, it has been revealed that some of these analogs display considerably stronger anticancer property than curcumin [50–53]. Hence, to overcome the restrictions of curcumin, researchers are developing and evaluating increasing number of curcumin novel analogs [166–168]. These analogs have shown a wide-spectrum of anticancer features in different cancer cells, such as colon [169], breast [170], and prostate [171].

Pan and colleagues indicated that, in plasma of curcumin-treated animals, 99% of the administered curcumin is glucuronide-conjugated [172]. It was also revealed that reduced curcumin metabolites, including curcumin glucuronosides, dihydrocurcumin and tetrahydrocurcumin are the main curcumin metabolites [172]. It has been shown that these metabolites have a crucial role in multiple therapeutic effects, including anticancer [173], anti-inflammation [174], and antioxidation [175]. However, numerous investigation have demonstrated that anticancer activity of curcumin metabolites is weaker in comparison to the parent molecule [176, 177]. Thus, several approaches, such as structural modification and improvising carrier molecules have been tested to compensate the curcumin's bioavailability limitations [176]. For increasing the therapeutic potential and bioavailability, different delivery systems including phospholipid complexes, micelles, nanoparticles, and liposomes have been recently recommended for curcumin treatment in cancer [176]. For instance, phospholipid complexes and micelles improve curcumin's gastrointestinal absorption, leading to greater plasma concentrations [176]. It has been revealed that curcumin-loaded amphiphilic mixed micelles has better bioavailability, causes remarkable accumulation of rhodamine, and modulates the expression levels of IL-6, IL-10 and TNF-α [41]. It also induced apoptosis and enhanced the intracellular uptake leading to inhibition of Ishikawa EC cells’ growth [41]. Xu et al. conducted a research to evaluate the efficacy of liposomal curcumin as a treatment for endometrial cancer [178]. Liposomal curcumin dose-dependently suppressed the motility, induced apoptosis and inhibited the proliferation of HEC-1 and Ishikawa cells [178]. It also blocked the expression of MMP-9, caspase-3, and NF-κB. Importantly, no toxicity was seen in the zebrafish model [178]. Demethoxycurcumin, a novel derivative of curcumin, induced apoptosis in cervical cancer cells *in vitro* and decreased tumor volume in an *in vivo* model [91]. In an investigation, curcumin-loaded chitosan nanoparticles were used for cervical cancer cells and it was shown that this delivery system enhanced the anti-proliferative effects of the drug against cancer cells and was not toxic to normal cells [90]. Du et al. reported that demethoxycurcumin, a curcumin analog, inhibited insulin receptor substrate–2 (IRS2) through miR-551a upregulation leading to hindering of ovarian cancer cells growth [134]. Compared to free curcumin, curcumin-loaded nanostructured lipid carriers (NLCs) showed better anticancer results, and more effectively reduced cell colony survival. These findings suggest that the curcumin entrapment into NLCs enhances the efficacy of curcumin *in vitro* [137]. Hosseini et al. have recently demonstrated that dendrosomal nanocurcumin (DNC) leads to greater cell death when combined with oxaliplatin. DNC also induced apoptosis in ovarian cancer cells [179]. De Matos et al. indicated that curcumin-nanoemulsion acts as a photosensitizing agent in PDT, demonstrating potential alternative capacity for cervical lesions [86]. Making novel formulations such as effective derivatives, as well as utilizing novel drug delivery systems can significantly increase the anticancer potential of curcumin. More experimental studies and human trials are needed to confirm the efficacy of curcumin and its novel formulations for the treatment of gynecological cancers.

## Conclusion and future perspectives

Gynecologic cancers represent a wide variety of cancers such as ovarian, uterine/endometrial, cervical, gestational trophoblastic, primary peritoneal, vaginal and vulvar cancers. Some of these cancers are known as “silent killer”, since the patient is usually unaware of tumor presence, until it is too late to cure. On the other hand, current therapeutic approaches carry serious limitations. Hence, developing new therapeutic platforms could optimize the treatment outcome for these cancers.

Curcumin is a well-known natural compound, which possesses a wide range of biologic effects such as anti-inflammatory, anti-cancer, and anti-oxidant activities. Several studies have proved the potential benefit of curcumin treatment both with monotherapy and in combination with standard chemotherapy drugs. The anti-cancer effects of curcumin in gynecologic cancers is shown to be linked to activation of autophagic and apoptotic pathways and inhibition of invasion and metastasis in various tumors. Curcumin suppresses major regulatory genes such as NF-κB and its downstream gene targets, which play significant roles in controlling invasion and metastasis of cancer cells. Interestingly, curcumin has been shown to improve the efficacy of current therapies by sensitizing resistance cancer cells to chemoradiotherapy, which has been a big obstacle in cancer treatment. Importantly, the safety and well-tolerability of curcumin has been demonstrated in clinical trials.

Along with different beneficial effects, curcumin treatment faces some limitations such as low *bioavailability*. However, several studies have proposed new formulations of curcumin to overcome this problem. Also utilization of different analogues and novel delivery systems such as nanoparticles, liposomes and micelles may further improve the anti-cancer effects of curcumin and open a new horizon in the treatment of gynecologic cancers.
